# Genetic Diversification and Population Admixture Signatures in Yunnan Native Cattle

**DOI:** 10.3390/ani16071105

**Published:** 2026-04-03

**Authors:** Yiduan Liu, Wenbin Dao, Wenkun Xu, Xinyang Fan, Ruifei Yang, Yongwang Miao

**Affiliations:** 1 Institute of Animal Genetics and Breeding, College of Animal Science and Technology, Yunnan Agricultural University, Kunming 650201, China; liuyiduanabc@163.com (Y.L.); dwbin666@126.com (W.D.); xinyangfan1@ynau.edu.cn (X.F.); 2 Animal Husbandry Station of Yunnan Province, Kunming 650224, China; xwk1974@126.com

**Keywords:** genetic diversity, population structure, Yunnan native cattle, balancing selection, positive selection, introgression

## Abstract

This study investigates the genetic diversity, population structure, and adaptive differentiation of Yunnan native cattle (YNC) using whole-genome SNP data from 457 individuals, representing eight cattle populations and two closely related bovine species (Zhongdian yak and Dulong gayal). Genetic diversity analyses revealed a distinct latitudinal gradient from north to south, with the highest diversity observed in the northern Diqing (DQC) and Zhaotong (ZTC) populations. The observed population structure was largely consistent with geographic distribution, identifying distinct ancestral components and complex admixture patterns. Genome-wide selective sweep scans revealed several key candidate genes underlying local adaptation. Notably, *GRIA4* and *DUOXA2* were associated with cold tolerance in northern populations, and *ST3GAL3* and *MST1* were implicated in heat stress adaptation in southern populations. Genome-wide balancing selection analyses further detected significant loci, such as *MGST1* and *SLC36A1*, where divergent haplotype frequencies reflected differential selective pressures on milk-related traits between northern and southern populations. Additionally, we detected signals of historical introgression from Zhongdian yak into DQC cattle, highlighting the introgressed gene *SLIT3* as a potential candidate associated with high-altitude thermogenesis. Collectively, these results provide a comprehensive genomic framework for the management and conservation of indigenous bovine genetic resources in Southwest China.

## Simple Summary

Yunnan Province in Southwest China serves as a critical biological corridor where diverse cattle lineages overlap. This study utilizes genome-wide single nucleotide polymorphism analysis to evaluate the genetic structure and environmental adaptation of eight native cattle breeds across this region. Our results identified a distinct north–south divide in genetic diversity and ancestral admixture. We identified specific genes responsible for cold adaptation in the highlands and heat tolerance in the tropical lowlands, as well as certain genetic variations under balancing selection. These findings reveal the genetic diversity and differentiation among Yunnan native cattle populations.

## 1. Introduction

The domestication and expansion of cattle are landmark events in human history, reflecting ancient migration routes and the development of agricultural trade networks. Genetic evidence indicates two primary domestication centers: approximately 10,500 years ago, *Bos taurus* was domesticated in the Fertile Crescent of the Near East, and around 8000 years ago, *Bos indicus* was domesticated in the Indus River basin [1,2]. Recent genomic studies have illuminated that domestic cattle in East Asia did not arise from a single origin but emerged through a gradual, multi-wave process marked by repeated external introductions and deep genetic integration with local aurochs (*Bos primigenius*) [3]. Around 4000–5000 years ago, taurine cattle entered Northeastern China, while zebu cattle reached the Central Plains of China around 3500 years ago via both inland and coastal routes through Southeast Asia, forming an admixture zone for taurine and zebu cattle, especially in Southwest China [3,4,5,6,7,8,9].

Yunnan Province, located at the junction of the Qinghai–Tibet Plateau and tropical Southeast Asia, features a remarkable ecological gradient from sub-frigid highlands to tropical rainforest. Yunnan currently recognizes eight native cattle breeds, each uniquely adapted to extreme environments through millennia of natural and artificial selection. For example, high-altitude Diqing cattle possess remarkable cold resistance, Dengchuan cattle are historically noted for high milk productivity, and southern Dehong humped cattle exhibit superior heat tolerance and parasite resistance. These cattle breeds are distributed along a north-to-south latitudinal gradient in Yunnan Province. Some studies have assessed the genetic structure of Yunnan native cattle (YNC), revealing a taurine–indicine admixture gradient across Yunnan [10,11,12]. The presence of related species like yak (*Bos grunniens*) and gayal (*Bos frontalis*) in the same region raises the possibility of interspecies introgression. However, the genomic mechanisms driving these extreme environmental adaptations remain poorly understood. Specifically, two fundamental scientific questions persist: how the long-term interaction between divergent taurine and zebu lineages, coupled with balancing selection, maintained the genetic heterogeneity observed along the latitudinal gradient; and how the introgression from related species such as yak (*Bos grunniens*) and gayal (*Bos frontalis*) contributed to adaptation and facilitated the rapid colonization of extreme niches.

We hypothesize that both ancient introgression and balancing selection have played pivotal roles in shaping this adaptive landscape. In this study, we utilize 100K genome-wide SNP data from 457 individuals to: (1) resolve the fine-scale population structure and ancestral components of YNC; (2) identify the genomic footprints of positive selection associated with cold and heat resilience as well as balancing selection, which maintains population diversification; and (3) quantify the adaptive impact of interspecies gene flow. These findings provide insights into the genetic landscape of YNC populations and establish a comprehensive SNP database, which is essential for preserving the genetic heritage of Southwestern Chinese cattle.

## 2. Materials and Methods

### 2.1. Sample Preparation and Genotyping Using SNP Chip Data

**Figure 1:**
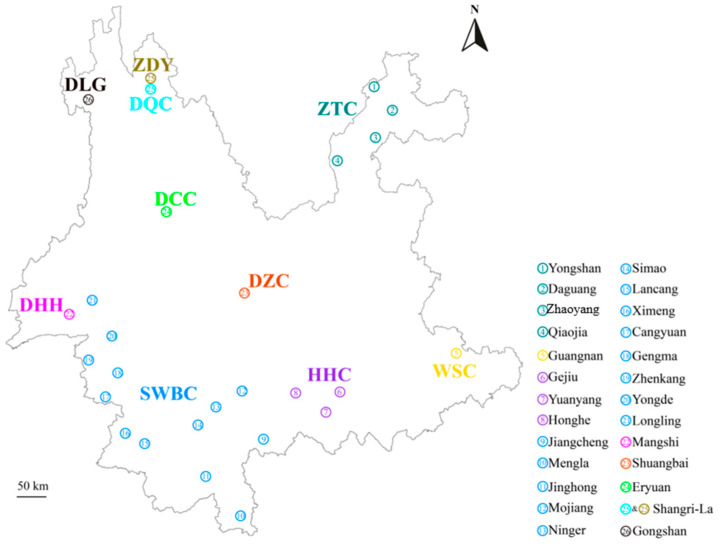
Geographical distribution of ten Yunnan native bovine populations. The sampling map illustrates 26 specific locations across Yunnan Province. Population abbreviations are defined as follows: Dulong gayal (DLG); Zhongdian yak (ZDY); Diqing cattle (DQC); Zhaotong cattle (ZTC); Dengchuan cattle (DCC); Dianzhong cattle (DZC); Dehong humped cattle (DHH); Cattle in the southwest border of Yunnan (SWBC); Honghe cattle (HHC); and Wenshan cattle (WSC).

Blood samples were collected from 457 individuals representing eight YNC breeds and two outgroup species (ZDY and DLG) across 26 sampling sites in Yunnan (Figure 1). The number of samples collected from each population and their collection locations are detailed in Table S1. To minimize kinship effects, we avoided sampling closely related individuals. Blood samples were collected via the jugular vein using EDTA anticoagulant tubes, stored at low temperatures, and transported to the laboratory. Whole genomic DNA was extracted using a magnetic bead-based kit (CWE9600 Magbead Blood DNA Kit, Kangwei Century, Taizhou, China). DNA quality and purity were determined via 0.8% agarose gel electrophoresis and ultraviolet spectrophotometry (NanoDrop 2000, Thermo Fisher Scientific, Waltham, MA, USA). Samples were genotyped using the GGP Bovine 100K Beadchip (Illumina Inc., San Diego, CA, USA), which includes over 101,220 SNPs anchored to the UMD3.1 assembly. To preserve the integrity of the original SNP coordinates and avoid data loss during genome liftover, all analyses in this study were performed using the UMD3.1 assembly. Key candidate regions and genes were further validated against the latest ARS-UCD2.0 assembly, confirming the consistency and reliability of our results. SNP quality control was performed using PLINK (v1.9) [13]. We retained only autosomal SNPs after filtering for MAF > 0.01 and a call rate >0.90 [14,15]. Beagle (v5.2) software was used to impute missing SNP genotypes [16]. The final dataset consisted of 457 individuals and 81,545 variants. The genotypic data are available in Dataset S1.

### 2.2. Phylogenetics and Population Structure

Following the calculation of identity-by-state (IBS) matrices using PLINK (v1.9; -distance square 1-ibs) [13], an unrooted neighbor-joining (NJ) tree was constructed with FastME (v2.1.6.1) [17] and visualized using the iTOL (v7.0) online [18]. For population structure analysis, SNP data were first pruned for linkage disequilibrium (LD) using PLINK’s ‘-indep-pairwise 50 10 0.2’ parameter. A threshold of *r*^2^ > 0.2 was chosen to balance the retention of genetic diversity and the reduction of redundant information due to high linkage disequilibrium, in line with recent approaches in selected cattle populations [19]. The LD-pruned data were then subjected to principal component analysis (PCA) via PLINK software and admixture analysis via ADMIXTURE (v1.3.0) [20] across K = 10, with the cross-validation error recorded for each K.

### 2.3. Population Genetic Diversity Analysis and Positive Selection Signals Detection

To assess genetic diversity across populations, PLINK software (v 1.9) was used to calculate minor allele frequency (MAF), observed heterozygosity (Ho), expected heterozygosity (He), and IBS genetic distance (D) [13,14]. VCFtools (v0.1.17) [21] was employed to calculate nucleotide diversity (Pi) within each population and F_ST_ between pairwise populations, with a window size of 50 kb. ROHs (Runs of Homozygosity) were identified using PLINK software (v 1.9), with a minimum ROH length set at 500 kb, each ROH containing at least 30 SNPs, and a minimum density of 100 kb/SNP [13,22,23]. ROHs were classified and statistically analyzed based on segment length: 0.5–1 Mb, 1–2 Mb, 2–4 Mb, 4–8 Mb, and ≥8 Mb.

Selection signals were analyzed by first calculating XPEHH scores with Selscan (v2.0.3) [24], which were normalized in 50 kb bins genome-wide. Given that the empirical distribution of XPEHH statistics approximates a normal distribution, we derived two-tailed *p*-values based on the standard normal distribution and considered values with *p* < 0.01 as significant outliers. In parallel, we calculated pairwise F_ST_ values using a 50 kb sliding window approach implemented in VCFtools [21]. Candidate regions under positive selection were defined as windows that concurrently met both criteria: an XPEHH-derived empirical *p*-value < 0.01 and an F_ST_ value falling within the top 5% of the genome-wide distribution.

### 2.4. Balancing Selection Analyses

To detect the genome traces of balancing selection, we used BetaScan2 (v1.0) [25] to calculate Beta1*Score with the parameter ‘-w 50,000 -fold’. Using VCFtools, nucleotide diversity (Pi) and Tajima’s D were calculated with a 50 kb sliding window [21]. We used PLINK software to calculate the linkage disequilibrium (LD) level (*r*^2^) between the top SNP with the highest Beta1*score with all flanking sites (500 kb), where the boundary of the haplotype was verified when LD was ≤0.4. Then, the haplotype was constructed based on the SNPs with Beta1*Score > 6 and LD *r*^2^ > 0.4. Finally, the haplotypes were retrieved from the different cattle groups and haplotype frequencies were calculated.

### 2.5. Global Ancestry and Local Ancestry Inference

To confirm the population admixture between ZDY and DQC and between DLG and WSC, we used *f*4 statistics and Treemix (v1.13) to identify global introgression signals [26,27]. For *f*4 statistics, we used the qpDstat program (v8.0.2) from the Admixture toolkit, with DLG and ZDY groups as the P1 and P2 groups; DQC, WSC, or other cattle populations (except ZTC) as P3; and ZTC as the P4 group. The |Z-score| of *f*4 statistics was used as evidence for significant gene flow between P2 and P3. For the Treemix analyses, we conducted five independent replicate runs to assess the stability of the results for each number of migration events (m ranging from 0 to 5). Model selection was performed using the OptM package (v0.1.9) with the linear method. Among the five replicates for each m value, we selected the representative run whose log-likelihood value was closest to the median of all replicates for subsequent visualization and interpretation. The SWBC population (n = 118) was considerably larger than the other populations, which could potentially influence the sensitivity or robustness of the *f*4-statistics and Treemix analyses. To evaluate whether this could lead to bias, we randomly subsampled 25 individuals from SWBC and re-ran the aforementioned analyses.

Based on the aforementioned inference of global ancestry, the local ancestry of the genome haplotypes was further inferred using RFmix software (v2.03) [28], which quantifies introgressive hybridization of genomic segments. For the RFmix analysis, the reference population (ZDY, DLG, and ZTC) consisted of genetic material from the target population (DQC or WSC). Considering non-overlapping haplotype blocks of 5 SNPs, a haplotype block was classified to originate from a specific population if its probability for that population exceeded 0.5. Specifically, each haplotype was encoded as ‘0’ if it was of ZTC cattle origin, or as ‘1’ if it originated from either ZDY or DLG. Further, we used VCFtools to compute the frequencies of introgressed haplotypes, and regions with haplotype frequencies exceeding 0.1 were considered as introgressed regions from ZDY or DLG. Tajima’s D and diversity Pi indicators were used to compare these regions against genome-wide levels.

## 3. Results

### 3.1. Genetic Diversity of Yunnan Native Cattle Populations

Our analysis revealed a clear latitudinal gradient in genetic diversity across Yunnan Province (Table 1). Compared to the domestic cattle populations, Dulong gayal (MAF = 0.129) and Zhongdian yak (MAF = 0.110) exhibited lower genetic diversity. Within domestic cattle populations, breeds located in the northern plateau (DQC and ZTC) exhibited higher levels of Ho and Pi compared to southern border populations (DHH and SWBC). Specifically, nucleotide diversity ranged from 1.487 × 10^−5^ in DQC to Pi = 0.941 × 10^−5^ in DHH. ROH analysis indicated varying levels of inbreeding and demographic history. A total of 20,339 ROHs were detected across genomic data from ten Yunnan *Bos* populations, with an average segment length of 3.74 Mb (Table 2). DLG and ZDY exhibited significantly longer ROH segments, consistent with their isolated and small population sizes [29]. DQC showed a high proportion of long ROH, while central breeds like DZC and HHC were dominated by medium segments (1–4 Mb), suggesting recent inbreeding or population bottlenecks.

### 3.2. Genetic Structure of Yunnan Native Cattle Populations

**Figure 2:**
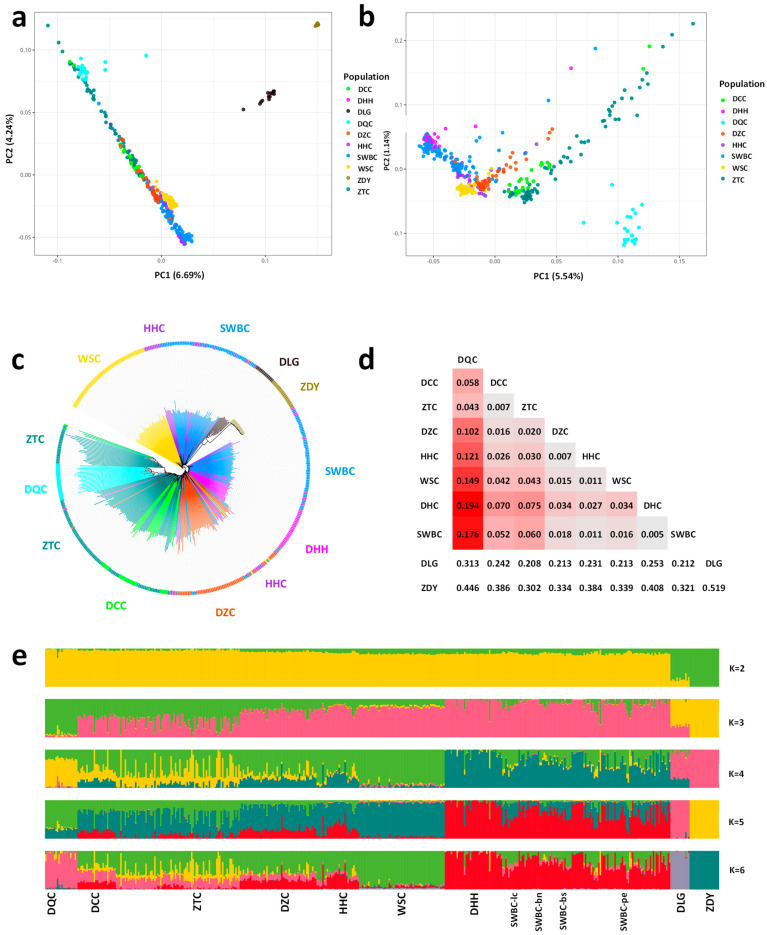
Population structure and genetic relationships of Yunnan native bovine populations. (**a**) Principal component analysis (PCA) of all ten populations, including yak and gayal. (**b**) PCA focused on the eight Yunnan native cattle (YNC) populations. (**c**) Neighbor-joining tree based on individual IBS genetic distances. (**d**) Heatmap of pairwise F_ST_ values representing genetic differentiation between YNC populations. (**e**) Admixture patterns inferred for K = 2 to 6; K = 6 represents the model with the lowest cross-validation error, highlighting the distinct ancestral components.

PCA and NJ tree analyses showed that YNC populations clustered mainly according to their geographic distribution along north–south and east–west gradients (Figure 1 and Figure 2). Figure 2b showed that PC1 and PC2 clearly distinguished DQC, ZTC, and DCC from other populations. Among the others, DZC, HHC, and WSC were clustered closely, and DHH and SWBC clustered together, which generally corresponded to a north-to-south and east-to-west geographical distribution of cattle populations in Yunnan Province. Furthermore, the NJ tree constructed based on the identity-by-state (IBS) matrix showed that DQC, DHH, WSC, and ZDC formed distinct clusters, whereas SWBC, HHC, and ZTC were each separated into two subclusters (Figure 2c). We computed the average F_ST_ values to compare the pairwise genetic differentiation in YNC populations. Apart from the outgroup, DQC showed the highest genetic differentiation compared to other cattle groups (F_ST_ ranging from 0.043 to 0.194) (Figure 2d), while the F_ST_ values between WSC, DHH, SWBC, DCC, and ZTC ranged from 0.04 to 0.07. To investigate the ancestry components in YNC populations, we conducted a population admixture analysis for the number of ancestors, i.e., K = 2 to 10 (Figure 2e and Figure S1). At K = 2, the outgroup, i.e., ZDY and DLG were distinguished from the cattle populations (Figure 2e). Based on the result for K = 6, which showed the lowest cross-validation error (CV = 0.496) (Figure S2), distinct ancestral components were identified for ZDY (99.9%), DLG (80.82–99.99%), DQC (54.02–97.78%), WSC (72.22–96.74%), and DHH (36.66–99.99%), while other cattle populations exhibited more admixed genetic backgrounds (Table S2). Southern breeds like SWBC showed high admixture between DHH and WSC components, reflecting gene flow across the southern border prefectures.

### 3.3. Positive Selection Analyses

DQC and ZTC exhibited the strongest genetic differentiation (Figure 2d) and were clearly separated in the PCA analysis (Figure 2b). As the northernmost breeds (Figure 1), they inhabit cold, dry climates, with DQC residing in the high-altitude Yunnan Plateau. By contrast, DHH was designated as the control population, and is distributed across the warmer southern border regions of Yunnan province (Figure 1 and Table S1).

**Figure 3:**
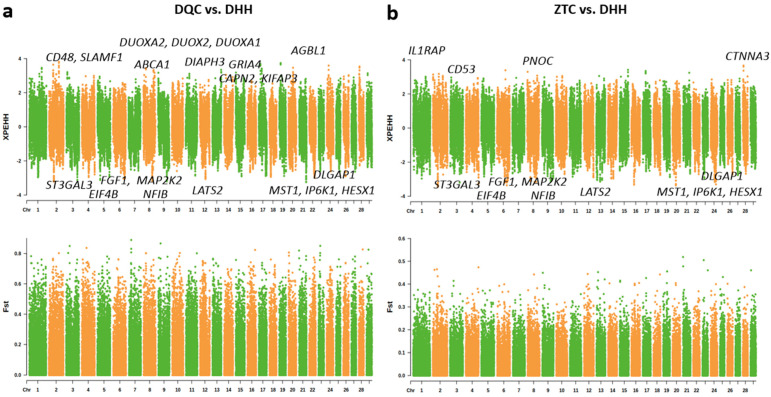
Manhattan plots of XPEHH and F_ST_ analyses for selective sweep detection. (**a**) Selective signals between DQC (Northern) and DHH (Southern) populations. (**b**) Selective signals between ZTC (Northern) and DHH (Southern) populations. Genes located within the overlapping outlier regions (the top 5% for F_ST_ and empirical *p* < 0.01 for XPEHH scores) are highlighted.

XPEHH and F_ST_ indicators were used to detect the signals of positive selection between the DQC and DHH populations, as well as between the ZTC and DHH populations. We detected 38 and 48 regions with significant positive selective signals in the DQC and ZTC breeds, respectively, corresponding to 26 and 27 genes under selection (Figure 3, Table S3, and Figure S3). By contrast, for the DHH breed, we detected 92 regions containing 62 genes with selective signals (Figure 3, Table S4, and Figure S3). Among these genes (Table S5), *GRIA4*, *DUOXA2*, *CD48*, and *SLAMF1*, associated with body thermoregulation and immunity, were selected in DQC [30,31,32,33,34,35,36]; *IL1RAP*, *CD53*, and *PNOC*, associated with cold stress and immune response, were selected in ZTC [37,38,39]; and *EIF4B* and *STAP2*, associated with heat stress and immune function, were selected in DHH [40,41]. Some genes involved in production were also detected, such as milk yield and composition (*DLGAP1*, *LATS2*, *MAP2K2*, *NFIB*, and *ABCA1*) [42,43,44,45,46,47], meat yield (*DIAPH3* and *CAPN2*) [48,49,50], tissue formation (*FGF1*) [51,52], growth (*HESX1*) [53], and bull fertility (*IP6K1*) [54].

### 3.4. Balancing Selection Analyses and Haplotype Comparison

**Figure 4:**
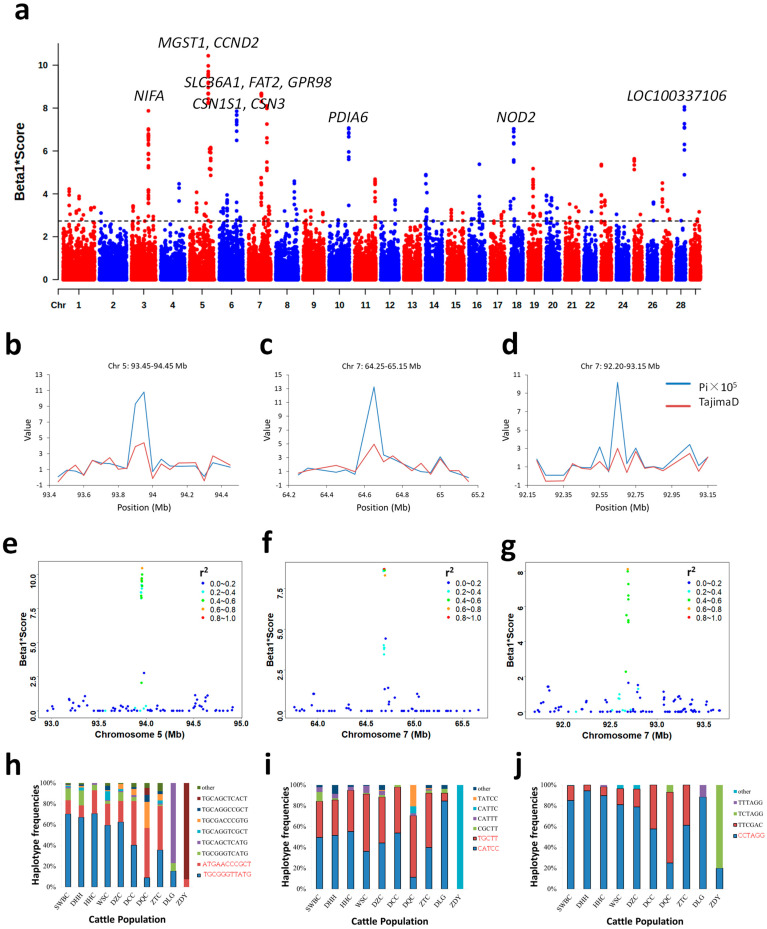
Balancing selection signals and haplotype frequency distributions. (**a**) Genome-wide Manhattan plot of balancing selection based on Beta1*Score. (**b**–**d**) Detailed regional scans of selective signals on chromosomes 5 and 7. (**e**–**g**) Correlation scatter plots between Beta1*Scores and LD *r*^2^ for candidate regions. (**h**–**j**) Haplotype frequency distributions across populations. Major haplotypes of functional interest are highlighted in red.

Balancing selection refers to the long-term maintenance of multiple alleles due to their bidirectional influence on fitness and is identified by a characteristic excess of polymorphisms at intermediate frequencies near the selected variant. Using BetaScan2 software on autosomal data, we identified 389 SNP loci under significant balancing selection (*FDR* < 1 × 10^−4^; Table S6). Among these, ten genomic regions exhibited extreme selection scores (Beta1*Score > 6; Figure 4a) and were consistently detected across most YNC subpopulations (Figure S4). To further validate these strong signals, we analyzed nucleotide diversity (Pi) and Tajima’s D within 50 kb sliding windows across ±500 kb flanking each region. These candidate regions displayed elevated Pi and positive Tajima’s D values compared to their genomic surroundings (Figure 4b–d and Figure S5), corroborating that they are indeed under balancing selection.

We next focused on the three regions exhibiting the most significant *FDR* values with the highest Beta1*Scores: chromosome 5: 93,942,166–93,954,751 bp (*MGST1* locus), chromosome 7: 64,679,944–64,695,210 bp (*SLC36A1* and *FAT2* loci), and chromosome 7: 92,688,306–92,693,936 bp (*GPR98* locus). Based on the top-scoring SNP in each region, LD analysis revealed that most linked loci (LD *r*^2^ > 0.4) also possessed extreme Beta1*Scores (>6) (Figure 4e–g). We therefore defined core haplotype blocks by integrating these two criteria (Beta1*Score > 6 and LD *r*^2^ > 0.4). Based on these defined blocks, we calculated the haplotype frequencies across all YNC subpopulations. For the *MGST1* locus on chromosome 5, most populations (except ZDY and DLG) exhibited two prevalent haplotypes: ‘TGCGGGTTATG’ (9.09–70.69%) and ‘ATGAACCCGCT’ (11.43–47.72%). Despite the presence of additional haplotypes, the combined frequency of these two major haplotypes was roughly equivalent to that of all other haplotypes combined in the DCC, DQC, and ZTC populations. For other cattle inhabited in the southwest area of Yunnan Province, ‘TGCGGGTTATG’ was the dominant haplotype in the populations of SWBC, DHH, HHC, WSC, and DZC (Figure 4h). For the *SLC36A1* and *FAT2* loci, DLG showed the highest frequency for haplotype ‘CATCC’ (84.62%). Excluding ZDY and DLG, all other groups maintained a relatively constant combined frequency for haplotype ‘CATCC’ (11.36–55.17%) or ‘TGCTT’ (34.29–59.09%) against the background of other haplotypes (Figure 4i). For the *GPR98* locus, only DCC, DQC, and ZTC maintained a stable frequency balance between haplotype ‘TTCGAC’ and all others (Figure 4j). The populations of SWBC, DHH, HHC, WSC, and DZC shared the highest-frequency haplotype ‘CCTAGG’, a pattern consistent with their Beta1*Score profiles (Figure 4j and Figure S4). Therefore, despite the detection of multiple strong signals of balancing selection in YNC populations, the haplotype frequencies underlying these signals exhibited substantial heterogeneity among different breeds and populations.

### 3.5. Analyses of ZDY- and DLG-Associated Ancestral Components in DQC and WSC Populations

**Figure 5:**
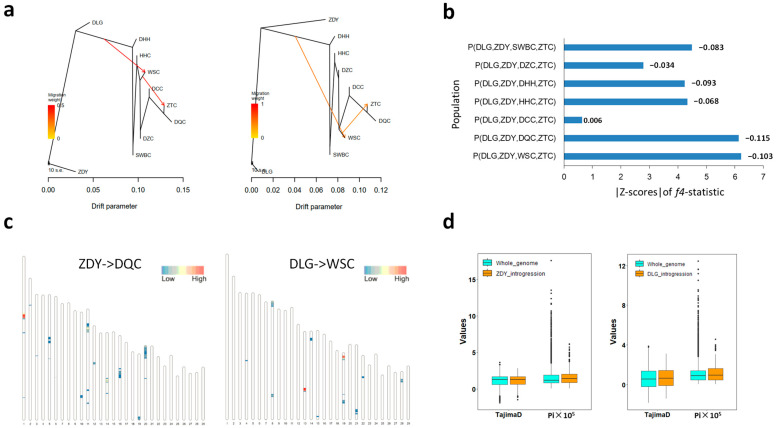
Population history and gene flow analyses of Yunnan bovine populations. (**a**) Maximum likelihood trees inferred via TreeMix analysis with two migration edges, using ZDY (left) and DLG (right) as outgroups. (**b**) Distribution of |Z-scores| from *f*4-statistics under the (DLG, ZDY; X, ZTC) topology; |Z| > 3 indicates significant gene flow. (**c**) Chromosomal distribution of ancestry regression coefficients for ZDY → DQC (left) and DLG → WSC (right) introgression events. (**d**) Comparison of nucleotide diversity (Pi) and Tajima’s D between introgressed segments and the whole-genome background.

As shown in Figure 2, ZDY and DLG constituted two independent ancestral genetic components, reflecting their distinct phylogenetic lineage compared to other populations. ADMIXTURE analysis at K = 6 revealed that DQC and WSC harbored some proportion of ancestry from these lineages, sharing approximately 0.1–28.3% and 1.2–5.4% ancestral components with ZDY and DLG, respectively. This pattern of shared ancestry was further supported by the haplotype analysis, which identified haplotypes characteristic of ZDY within DQC (Figure 4h–j). To confirm these historical introgression events, we performed Treemix analyses using ZDY or DLG as the outgroup, respectively. The Treemix analysis (m = 2) revealed a significant gene flow event into the WSC population (Figure 5a). The source of this migration was placed on a branch within the clade that included all other YNC populations, after the divergence of the distinct ZDY/DLG lineage. This topology indicated that the introgressing ancestry was derived not from the common ancestor of all populations but from an early-diverging sub-lineage within the mainstream YNC groups, subsequent to the evolutionary separation of the ZDY/DLG clade (Figure 5a and Figure S6). Furthermore, the more complex Treemix model (m = 5) revealed a direct introgression signal from ZDY into DQC (Figure S7), which was independently supported by significant f4-statistics under the (P (DLG, ZDY, DQC, ZTC)) topology (|Z-score| = 6.115) (Figure 5b). For WSC, the f4-statistics (P (DLG, ZDY, WSC, ZTC)) yielded a significantly negative value (|Z-score| = 6.103), demonstrating greater allele sharing with ZDY than with DLG. Integrating this with the ancient introgression into WSC inferred by the Treemix analyses (m = 2) supported a model in which the allele-sharing pattern was likely shaped by subsequent incomplete lineage sorting (ILS) of the ancestral variation introduced by this event.

To further infer the origin of the ancestry segments, we performed local ancestry inference using RFmix with ZDY, DLG, and ZTC as reference populations. The analysis revealed strikingly distinct patterns between the two recipient populations. DQC showed no detectable genomic segments from DLG but carried 390 segments assigned to ZDY ancestry, providing clear evidence of introgression from ZDY (Figure 5c and Table S7). By contrast, WSC carried only 7 segments from ZDY but 217 from DLG (Figure 5c and Table S7), indicating that a substantial proportion of the WSC genome was derived from gene flow originating in the DLG lineage. We then examined the selective landscape of the introgressed segments. Scans for positive selection (XPEHH and F_ST_ against ZTC) yielded few signals (7 windows in DQC, 1 in WSC; Table S8). Scans for balancing selection (BetaScan2) within the introgressed segments identified a small number of significant SNP loci with weak signals (Table S9). Compared to the genome-wide background, the ZDY-derived segments in DQC showed significantly elevated nucleotide diversity (Pi; *p*-value = 2.042 × 10^−7^) but not an elevated Tajima’s D (*p*-value = 0.122). In WSC, the DLG-derived segments exhibited significant elevations in both Pi (*p*-value = 1.821 × 10^−3^) and Tajima’s D (*p* = 0.038) (Figure 5d).

## 4. Discussion

### 4.1. Genetic Diversity and Evolutionary History of YNC

The higher heterozygosity and nucleotide diversity observed in northern populations (DQC and ZTC) likely reflect a combination of historical admixture, broader effective population sizes, and reduced directional selection compared with southwestern border breeds (DHH and SWBC). By contrast, the lower diversity detected in gayal and yak populations may be attributable to long-term geographic isolation and species-specific demographic constraints. This pattern is consistent with previous comparative studies in other bovine species [55,56]. Slight variations in heterozygosity values across different SNP chips have been reported [57,58,59,60], which are likely attributable to differences in chip design and sampling strategies. Overall, the genetic diversity of YNC shows a clear decreasing gradient from north to south, in agreement with earlier findings [12,14]. Despite their relatively high genetic diversity, the presence of long runs of homozygosity (ROH) in Diqing cattle suggests recent inbreeding or localized population bottlenecks, potentially driven by geographic isolation in the high-altitude northwestern mountains. Similar to previous reports, the observed heterozygosity (Ho) values were consistently lower than the expected heterozygosity (He) in local cattle populations, indicating a decline in genetic diversity [12,14]. This reduction highlights the need for improved breeding management. Notably, resequencing data have shown that, compared with commercial breeds, Yunnan indigenous cattle exhibit higher levels of nucleotide diversity [11].

PCA and NJ tree analyses revealed a north-to-south geographic pattern in the genetic structure of YNC populations (Figure 2). DQC were clearly separated from other populations in both analyses, reflecting their unique ancestral composition and geographic isolation in the high-altitude northwestern boundary region of Yunnan Province. While our current dataset focuses on Yunnan populations, the independent ancestral component in DQC likely derives from taurine-related ancestry. Future analyses incorporating broader global reference panels will further clarify this lineage. This interpretation is supported by previous work indicating that the taurine lineage of Diqing cattle is primarily of East Asian taurine origin [11]. WSC and DHH each formed distinct ancestral components. Several other populations shared genetic components with these breeds: WSC-derived components were detected in DCC, DZC, HHC, and ZTC, whereas a DHH-derived component was present in SWBC (Figure 2e). This pattern of shared ancestry corresponds to an east-to-west geographic distribution (Figure 1). We speculate that these components may originate from two distinct zebu cattle sources. Specifically, Indian zebu cattle from South Asia may have entered Yunnan Province through border regions such as Dehong Prefecture, whereas eastern regions such as Wenshan Prefecture may represent a primary breeding center for Chinese zebu cattle. The discontinuous distribution of these two zebu lineages is likely shaped by geographic barriers, including rivers and mountain ranges. We also observed within-breed population substructure in ZTC (Figure 2). As this population was sampled from four distinct areas in the northeastern boundary region of Yunnan Province, the observed substructure likely reflects local adaptation or differential admixture. Some individuals may also harbor ancestry from cattle breeds distributed in other regions worldwide. According to local records, the use of frozen Simmental semen for artificial insemination to improve production traits is widespread in Zhaotong Prefecture. In addition, a ZTC-like component was detected in DCC (14.72%), a breed known for milk production and historically crossbred with Dutch bulls since 1954 [29]. We therefore speculate that this component in ZTC and DCC likely represents introgression from European cattle breeds. Further studies incorporating a broader panel of global cattle populations will be required to fully resolve this substructure. Among the populations located in central Yunnan Province, DZC exhibited multiple genetic components derived from other cattle breeds (Figure 1 and Figure 2). Overall, YNC appear to represent an admixed population with a complex genetic background derived from *Bos taurus* and two distinct zebu ancestries. The diverse climatic and geographical landscapes of Yunnan Province likely promoted and maintained the high level of genetic diversity observed in YNC populations.

### 4.2. Positive and Balancing Selection in YNC Populations

In this study, we first conducted genome-wide scans for positive selection across YNC populations. Among them, DQC, ZTC, and DHH were selected for comparative analysis as representative breeds inhabiting the northern and southern boundary regions of Yunnan Province, respectively. In ZTC and DQC, we identified selection signatures in genes associated with cold tolerance and high-altitude adaptation, including *GRIA4* and members of the NADPH oxidase family (*DUOX2*, *DUOXA2*, and *DUOXA1*), which are involved in body thermoregulation and energy metabolism. A novel nonsynonymous SNP in *DUOXA2* has been reported to differ between Tibetan taurine cattle and other cattle populations and to be involved in the thyroid hormone synthesis pathway [30]. *DUOXA2* aids *DUOX2* in migrating from the endoplasmic reticulum to the plasma membrane, and their complex promotes H_2_O_2_ generation in thyroid hormone synthesis, while *DUOXA2* mutations disrupt this process [61]. The *DUOXA1/DUOX1* system performs similar functions [62]. Seasonal variations in thyroid hormones are influenced by winter temperatures. Given their key role in nonshivering thermogenesis, these variations may reflect differences in thyroid hormone utilization in brown adipose tissue during adaptive thermogenesis, depending on the intensity of cold exposure [63]. *GRIA4*, which encodes glutamate ionotropic receptor AMPA type subunit 4, has been recognized as a candidate gene involved in body temperature maintenance under cold stress in Siberian cattle [31,32]. In response to hypothermia, animals mainly activate thermogenic and thermoregulatory mechanisms [64]. These processes are initiated by the detection of thermal stimuli via thermoreceptors and the subsequent integration of thermal signals in the hypothalamic center [64]. Functionally, *GRIA4* is strongly associated with cell adhesion, calcium ion binding, and synapses, and it exhibits high expression in the brain [65]. Additionally, *GluK2*, another glutamate receptor, has been identified to mediate synaptic transmission in the brain and participate in cold temperature sensing in mouse peripheral neurons [66,67]. In addition, *AGBL1*, which is involved in adipogenic differentiation [68,69], may contribute to the regulation of energy metabolism in cold environments. By contrast, selection signals detected in DHH are associated with different phenotypic traits, particularly genes related to cell signaling and immune responses, such as *MST1* and *ST3GAL3*. *MST1* functions as a regulator of the Hippo pathway in immune system [70,71,72]. The Hippo kinase genes (*MST1* and *MST2*) play key roles in host defense against microbial infection and regulate immune activities at multiple levels in macrophages, including gene expression, immune cell communication, and programmed cell death [73]. *ST3GAL3* has been shown to positively affect heat tolerance in Egyptian sheep [74], and loss of the *ST3GAL3* gene reduces cell proliferation and suppresses AKT phosphorylation [75]. These findings suggest that *ST3GAL3* may be involved in heat stress responses, potentially through the modulation of inflammatory signaling pathways. Collectively, DHH populations may adapt to tropical environments by enhancing heat tolerance and strengthening innate immune responses against parasites in tropical lowlands.

Multiple genomic regions under balancing selection were identified in YNC (Figure 4a). These included loci such as *NIFA* on chromosome 3, as well as genes associated with production traits (*CSN1S1*, *CSN3*, *CCND2*, and *PDIA6*) [76,77,78,79] and immune-related traits (*NOD2*) [80]. The most prominent balancing selection signals were detected at the *MGST1* locus on chromosome 5 and the *SLC36A1_FAT2* and *GPR98* loci on chromosome 7. Across these three loci, haplotypes exhibited opposing frequency patterns corresponding to the north–south geographic distribution of YNC populations (Figure 4h–j). At the *MGST1* locus, the haplotype ‘TGCGGGTTATG’ was most frequent in southern and central populations (SWBC, DHH, HHC, WSC, and DZC), whereas ‘ATGAACCCGCT’ was dominant in northern populations (DCC, DQC, and ZTC) (Figure 1 and Figure 4h). Similar north–south divergence patterns were observed at the *SLC36A1_FAT2* and *GPR98* loci on chromosome 7 (Figure 1, and Figure 4i,j). Furthermore, while several balancing selection signals were identified across the entire YNC population, the evolutionary “balanced” state may not be uniformly maintained across all subsets. Notably, although balancing selection signals were detected at the *GPR98* locus across the entire YNC population, the haplotypes ‘CCTAGG’ and ‘TTCGAC’ displayed remarkable frequency differences between northern and southern groups (Figure 4j), reflecting their distinct ancestral origins. The ‘CCTAGG’ haplotype was predominant in southern populations, suggesting that local adaptation or differential selective pressures across geographic regions may have shaped the haplotype distribution of this locus, despite the locus appearing “balanced” when viewing the YNC population as a whole. Functionally, *MGST1* is strongly associated with milk fat content [81], which is consistent with the long-standing historical tradition of cattle being raised for milk production in northwestern Yunnan (such as in Diqing and Dali Prefectures), whereas southern populations are mainly kept for draft and meat production [29]. As early as 1796, local farmers in Dali Prefecture began producing a traditional regional dairy product by concentrating fresh milk into a solid form [29]. Several candidate genes associated with milk yield and composition have been reported, including *ITSN2* (related to environmental mastitis), *B4GALT1*, *RERE*, and *SLC45A1* (associated with milk production), as well as *MTERF3* (linked to milk fatty acid composition) [82]. *SLC36A1* is involved in milk protein synthesis in yak [83,84], and historical records indicate that DQC hybridized with yak, giving rise to high-milk-yielding Cattle-Yak [29]. Whey proteins and bioactive peptides derived from Cattle-Yak show greater diversity and higher activity compared to those derived from Zhongdian yak and Diqing cattle [85]. *FAT2* and *GPR98* are implicated in diverse biological processes, including regulation of cell migration [86], motor behavior in mice [87], and cell–cell or cell–matrix interactions [88], while their roles in cattle remain poorly understood and warrant further investigation. Although these loci were identified as targets of balancing selection across YNC populations, their haplotype distributions primarily reflect genetic differentiation between northern and southern cattle groups. However, the absence of balancing selection signals in some breeds may be attributable to the limited resolution of SNP chip data, as its lower marker density (~1 SNP/32.7 Kb) is insufficient to capture rapid LD decay patterns. Genome resequencing data would therefore be necessary to validate whether these missing signals reflect true biological absence or technical artifacts.

### 4.3. Possible Introgression Patterns of ZDY and DLG into DQC and WSC and Their Selection Analyses

Population admixture analyses revealed ancestral components derived from Zhongdian yak and Dulong gayal in DQC and WSC, respectively. These signals were further confirmed as historical introgression events through *f*4-statistics and Treemix analyses (Figure 2e, Figure 5b and Figure S7). To assess the robustness of these findings, we randomly down-sampled the SWBC population (n = 25) and re-ran the *f*4-statistics and Treemix analyses. The results consistently confirmed the global introgression signals observed in the full dataset (Figures S8–S10). Introgression from ZDY into DQC appears to be a relatively recent event, supported by some individuals with high proportions of ZDY ancestry (10.6–28.3%) and by historical records documenting hybridization since the late twentieth century, consistent with geographic proximity and mixed husbandry practices (Figure 1). Bidirectional genetic introgression between Diqing cattle and Zhongdian yak has also been reported previously [89]. By contrast, the DLG-related ancestry observed in WSC reflects a more complex and ancient process. The Treemix analysis did not infer direct gene flow from DLG into WSC but rather from an ancestral population that diverged after the ZDY/DLG split (Figure 5a), implying that DLG introgression into WSC may have been mediated by this ancestor. Similarly, *f*4-statistics analysis did not detect a direct DLG-to-WSC signal but instead indicated allele sharing between ZDY and WSC (Figure 5b). Previous studies have identified introgressed genomic regions from Yunnan gayal into Yunnan domestic cattle and southern Chinese cattle [11,60], including genes related to heat stress, such as *HSPBAP1* [11]. Genetic ancestries from other related bovine species, including banteng and gaur, have also been detected in East Asian indicine cattle, particularly in southeastern coastal regions of China [9,90]. We therefore speculate that Wenshan cattle may represent a historical hybrid derived from multiple closely-related bovine species, some of which may no longer be locally present. This admixture history likely predates their arrival in Wenshan Prefecture, as no current records indicate the presence of banteng or gaur in the region.

RFmix analyses quantifying genome-wide introgression further indicated that DQC was primarily introgressed from ZDY, while WSC carried a higher proportion of DLG-derived haplotypes, consistent with the admixture results (Figure 2e). Scans for positive and balancing selection identified candidate genes within introgressed regions. In DQC, the genes located in positively selected regions included *SIDT1*, *SCYL3*, *KIFAP3*, and *SLIT3*, whereas in WSC, *ZFAT* was the only gene detected near a selection signal. *ZFAT* functions as a critical transcriptional regulator in B and T lymphocytes [91], while *KIFAP3* has been associated with temperament traits, particularly flight time [92]. The identification of *SLIT3* within introgressed segments is of particular interest. This gene is increasingly recognized for its role in adaptive thermogenesis, a key physiological process for cold adaptation. In mammals, brown adipose tissue (BAT) is central to adaptive thermogenesis. Recent mouse studies have shown that *SLIT3* is involved in a signaling pathway that regulates BAT function and long-term cold adaptation [93]. Specifically, *SLIT3* acts as a key mediator in the macrophage–Slit3–sympathetic neuron–adipocyte signaling axis, which modulates BAT thermogenesis [93]. It also promotes crosstalk among adipocyte progenitors, angiogenesis, and sympathetic innervation, all of which are essential for BAT thermogenic activity in vivo [94]. Additionally, cell heterogeneity in adipose tissues has been identified in Mongolian cattle [95]. This finding suggests potential species-specific regulatory patterns of adipocyte-related signaling pathways in cattle, which may involve *SLIT3*-mediated thermogenic regulation. In contrast to its well-characterized functions in model organisms, research on *SLIT3* in cattle and yak remains limited. Existing reports are scarce and primarily link *SLIT3* to phenotypic traits such as bone formation and tail length in Simmental cattle [96], as well as hair length and reproductive cycles in yak [97,98]. Moreover, the methylation level of the *SLIT3* exon region in the spleen is higher in Yunnan zebu cattle than in Holstein cattle [99]. However, no experimental validation has confirmed its specific regulatory mechanisms for these traits. Importantly, its role in thermogenesis and cold adaptation has not yet been explored in cattle or yak. Given its known role in modulating adipocyte signaling and BAT function in model organisms, *SLIT3* is a strong candidate gene for cold adaptation in DQC cattle. It may contribute to this trait by regulating adipocyte-mediated thermogenesis. Therefore, future research should focus on the functional validation of *SLIT3* in bovine species. Balancing selection analyses revealed that only a small number of SNPs within introgressed regions exhibited significant but relatively weak signals in BetaScan2 analyses (Tables S8 and S9). Introgressed segments in both DQC and WSC showed stronger balancing selection intensities than the genome-wide background, with DLG-derived segments in WSC exhibiting particularly strong signals (Figure 5d). However, most introgressed genomic segments lacked strong positive or balancing selection signals. This absence of pronounced selective signatures is consistent with a complex admixture history. Rather than reflecting a simple adaptive introgression model characterized by strong selective sweeps, our results are more consistent with neutral or background introgression. Introgressed haplotypes may have interacted with local genetic or environmental contexts, leading to frequency differences between northern and southern populations through genetic drift or weak, polygenic selection rather than through strong, single-gene sweeps. Thus, introgressed segments from ZDY into DQC and from DLG into WSC appear to persist largely due to neutral demographic processes or weak polygenic adaptation.

## 5. Conclusions

In conclusion, our population genetic analyses revealed that the genetic diversity and phylogenetic structure of YNC closely correspond to their north–south geographic distribution. We identified critical adaptive genes, including *GRIA4* and *DUOXA2*, which are associated with cold adaptation, and *ST3GAL3* and *MST1*, which are related to heat resilience. Additionally, we detected strong balancing selection signals at loci such as *MGST1*, *SLC36A1_FAT2*, and *GPR98*, which reflect significant genetic differentiation and distinct agricultural trajectories between northern and southern cattle groups. Furthermore, we provide robust genetic evidence confirming historical introgression from Zhongdian yak into DQC and from the Dulong gayal lineage into WSC. The lack of strong positive or balancing selection signals in most introgressed segments suggests that their persistence may be driven primarily by neutral demographic processes or weak polygenic adaptation. Nevertheless, interspecies gene flow has introduced potentially adaptive alleles, such as *SLIT3*, into high-altitude cattle populations. Collectively, these findings illuminate the unique and complex genetic architecture of YNC populations, which has been intricately shaped by diverse climatic conditions and a dynamic admixture history within Southwest China.

## Figures and Tables

**Table 1:** Genetic diversity metrics estimated within each population.

Population	Sample Size	Cleaned SNPs	^1^ MAF	^2^ Ho	^3^ He	^4^ Pi (×10^−5^)	^5^ D
DQC	22	82,903	0.281	0.367	0.366	1.487	0.300
DCC	26	83,306	0.243	0.328	0.330	1.337	0.274
ZTC	84	85,418	0.259	0.340	0.348	1.407	0.285
DZC	52	80,971	0.215	0.283	0.301	1.166	0.255
HHC	29	79,203	0.202	0.269	0.284	1.118	0.244
WSC	58	77,407	0.194	0.273	0.275	1.050	0.229
DHH	35	77,490	0.169	0.239	0.241	0.941	0.204
SWBC	118	80,024	0.180	0.252	0.261	1.007	0.217
DLG	13	39,796	0.129	0.202	0.199	0.610	0.180
ZDY	20	9440	0.110	0.175	0.165	0.451	0.141

^1^ MAF, minor allele frequency; ^2^ Ho, observed heterozygosity; ^3^ He, expected heterozygosity; ^4^ Pi, nucleotide diversity; ^5^ D, average pairwise genetic distance calculated by 1—DST.

**Table 2:** ROH ranges in eight cattle breeds and two other bovine populations.

Population	No.	Total Length (Mb)	Total Number	Mean Length per Individual (Mb)	Average Number per Individual	Total Number of ROH (%)				
						500 kb to 1 Mb	1 Mb to 2 Mb	2 Mb to 4 Mb	4 Mb to 8 Mb	≥8 Mb
DQC	22	2285.50	384	103.89	17	2.86	27.86	29.43	20.05	19.79
DCC	26	2859.99	712	110.00	27	4.49	54.35	27.39	4.92	8.85
ZTC	84	7910.47	2189	94.17	26	4.2	57.20	26.18	5.25	7.17
DZC	52	11,818.34	2303	227.28	44	2.43	47.50	28.70	7.73	13.63
HHC	29	6979.63	1330	240.68	46	2.63	40.53	35.11	8.35	13.38
WSC	58	6764.49	2826	116.63	49	2.58	49.65	42.92	3.43	1.42
DHH	35	4471.09	1797	127.75	51	2.73	53.09	38.56	3.01	2.62
SWBC	118	19,495.01	6433	165.21	55	3.02	53.26	35.24	3.64	4.85
DLG	13	10,159.92	1686	781.53	130	0	3.56	38.73	38.85	18.86
ZDY	20	3385.81	679	169.29	34	0.15	5.15	38.73	44.04	11.93

## Data Availability

The genotype data presented in this study are included in the Supplementary Material. Further inquiries can be directed to the corresponding authors.

## Supplementary Materials

The following supporting information can be downloaded at: https://www.mdpi.com/article/10.3390/ani16071105/s1.

## References

[1] Achilli A., Olivieri A., Pellecchia M., Uboldi C., Colli L., Al-Zahery N., Accetturo M., Pala M., Hooshiar Kashani B., Perego U.A. (2008). Mitochondrial Genomes of Extinct Aurochs Survive in Domestic Cattle. Curr. Biol..

[2] Chen S., Lin B.-Z., Baig M., Mitra B., Lopes R.J., Santos A.M., Magee D.A., Azevedo M., Tarroso P., Sasazaki S. (2010). Zebu Cattle Are an Exclusive Legacy of the South Asia Neolithic. Mol. Biol. Evol..

[3] Cai D., Kim D., Zhang N., Wang X., Li T., Li J., Li C., Lian S., Shao X., Hu S. (2025). Ancient Genomes Illuminate the Origins and Dynamic History of East Asian Cattle. Science.

[4] Chen N., Cai Y., Chen Q., Li R., Wang K., Huang Y., Hu S., Huang S., Zhang H., Zheng Z. (2018). Whole-Genome Resequencing Reveals World-Wide Ancestry and Adaptive Introgression Events of Domesticated Cattle in East Asia. Nat. Commun..

[5] Cai Y., Jiao T., Lei Z., Liu L., Zhao S. (2018). Maternal Genetic and Phylogenetic Characteristics of Domesticated Cattle in Northwestern China. PLoS ONE.

[6] Cai D., Sun Y., Tang Z., Hu S., Li W., Zhao X., Xiang H., Zhou H. (2014). The Origins of Chinese Domestic Cattle as Revealed by Ancient DNA Analysis. J. Archaeol. Sci..

[7] Zhang W., Gao X., Zhang Y., Zhao Y., Zhang J., Jia Y., Zhu B., Xu L., Zhang L., Gao H. (2018). Genome-Wide Assessment of Genetic Diversity and Population Structure Insights into Admixture and Introgression in Chinese Indigenous Cattle. BMC Genet..

[8] Utsunomiya Y.T., Milanesi M., Fortes M.R.S., Porto-Neto L.R., Utsunomiya A.T.H., Silva M.V.G.B., Garcia J.F., Ajmone-Marsan P. (2019). Genomic Clues of the Evolutionary History of *Bos indicus* Cattle. Anim. Genet..

[9] Chen N., Xia X., Hanif Q., Zhang F., Dang R., Huang B., Lyu Y., Luo X., Zhang H., Yan H. (2023). Global Genetic Diversity, Introgression, and Evolutionary Adaptation of Indicine Cattle Revealed by Whole Genome Sequencing. Nat. Commun..

[10] Li R., Li C., Liu H., Zeng B., Xiao H., Chen S. (2018). Mitochondrial Diversity and Phylogeographic Structure of Native Cattle Breeds from Yunnan, Southwestern China. Livest. Sci..

[11] Guan X., Xiang W., Qu K., Ahmed Z., Liu J., Cai M., Zhang J., Chen N., Lei C., Huang B. (2025). Whole Genome Insights into Genetic Diversity, Introgression, and Adaptation of Yunnan Indigenous Cattle of Southwestern China. BMC Genom..

[12] Li R., Li C., Chen H., Liu X., Xiao H., Chen S. (2019). Genomic Diversity and Admixture Patterns among Six Chinese Indigenous Cattle Breeds in Yunnan. Asian-Australas. J. Anim. Sci..

[13] Purcell S., Neale B., Todd-Brown K., Thomas L., Ferreira M.A.R., Bender D., Maller J., Sklar P., de Bakker P.I.W., Daly M.J. (2007). PLINK: A Tool Set for Whole-Genome Association and Population-Based Linkage Analyses. Am. J. Hum. Genet..

[14] Freitas P.H.F., Wang Y., Yan P., Oliveira H.R., Schenkel F.S., Zhang Y., Xu Q., Brito L.F. (2021). Genetic Diversity and Signatures of Selection for Thermal Stress in Cattle and Other Two Bos Species Adapted to Divergent Climatic Conditions. Front. Genet..

[15] Xu L., Yang L., Zhu B., Zhang W., Wang Z., Chen Y., Zhang L., Gao X., Gao H., Liu G.E. (2019). Genome-Wide Scan Reveals Genetic Divergence and Diverse Adaptive Selection in Chinese Local Cattle. BMC Genom..

[16] Browning B.L., Tian X., Zhou Y., Browning S.R. (2021). Fast Two-Stage Phasing of Large-Scale Sequence Data. Am. J. Hum. Genet..

[17] Lefort V., Desper R., Gascuel O. (2015). FastME 2.0: A Comprehensive, Accurate, and Fast Distance-Based Phylogeny Inference Program. Mol. Biol. Evol..

[18] Letunic I., Bork P. (2007). Interactive Tree Of Life (iTOL): An Online Tool for Phylogenetic Tree Display and Annotation. Bioinformatics.

[19] Bordbar F., Jensen J., Wadood A.A., Yao Z. (2024). Linkage Disequilibrium Decay in Selected Cattle Breeds. Animals.

[20] Alexander D.H., Novembre J., Lange K. (2009). Fast Model-Based Estimation of Ancestry in Unrelated Individuals. Genome Res..

[21] Danecek P., Auton A., Abecasis G., Albers C.A., Banks E., DePristo M.A., Handsaker R.E., Lunter G., Marth G.T., Sherry S.T. (2011). The Variant Call Format and VCFtools. Bioinformatics.

[22] Xu L., Zhao G., Yang L., Zhu B., Chen Y., Zhang L., Gao X., Gao H., Liu G.E., Li J. (2019). Genomic Patterns of Homozygosity in Chinese Local Cattle. Sci. Rep..

[23] Dixit S.P., Singh S., Ganguly I., Bhatia A.K., Sharma A., Kumar N.A., Dang A.K., Jayakumar S. (2020). Genome-Wide Runs of Homozygosity Revealed Selection Signatures in *Bos indicus*. Front. Genet..

[24] Szpiech Z.A., Hernandez R.D. (2014). Selscan: An Efficient Multithreaded Program to Perform EHH-Based Scans for Positive Selection. Mol. Biol. Evol..

[25] Siewert K.M., Voight B.F. (2020). BetaScan2: Standardized Statistics to Detect Balancing Selection Utilizing Substitution Data. Genome Biol. Evol..

[26] Patterson N., Moorjani P., Luo Y., Mallick S., Rohland N., Zhan Y., Genschoreck T., Webster T., Reich D. (2012). Ancient Admixture in Human History. Genetics.

[27] Pickrell J.K., Pritchard J.K. (2012). Inference of Population Splits and Mixtures from Genome-Wide Allele Frequency Data. PLoS Genet..

[28] Maples B.K., Gravel S., Kenny E.E., Bustamante C.D. (2013). RFMix: A Discriminative Modeling Approach for Rapid and Robust Local-Ancestry Inference. Am. J. Hum. Genet..

[29] Yunnan Provincial Livestock and Poultry Genetic Resources Committee (2014). Livestock and Poultry Genetic Resources in Yunnan Province.

[30] Lyu Y., Wang F., Cheng H., Han J., Dang R., Xia X., Wang H., Zhong J., Lenstra J.A., Zhang H. (2024). Recent Selection and Introgression Facilitated High-Altitude Adaptation in Cattle. Sci. Bull..

[31] Igoshin A.V., Yurchenko A.A., Belonogova N.M., Petrovsky D.V., Aitnazarov R.B., Soloshenko V.A., Yudin N.S., Larkin D.M. (2019). Genome-Wide Association Study and Scan for Signatures of Selection Point to Candidate Genes for Body Temperature Maintenance under the Cold Stress in Siberian Cattle Populations. BMC Genet..

[32] Igoshin A., Yudin N., Aitnazarov R., Yurchenko A.A., Larkin D.M. (2021). Whole-Genome Resequencing Points to Candidate DNA Loci Affecting Body Temperature under Cold Stress in Siberian Cattle Populations. Life.

[33] Lee S., Clémentine C., Kim H. (2024). Exploring the Genetic Factors behind the Discrepancy in Resistance to Bovine Tuberculosis between African Zebu Cattle and European Taurine Cattle. Sci. Rep..

[34] Sun L., Qu K., Liu Y., Ma X., Chen N., Zhang J., Huang B., Lei C. (2023). Assessing Genomic Diversity and Selective Pressures in Bashan Cattle by Whole-Genome Sequencing Data. Anim. Biotechnol..

[35] Xia X., Zhang S., Zhang H., Zhang Z., Chen N., Li Z., Sun H., Liu X., Lyu S., Wang X. (2021). Assessing Genomic Diversity and Signatures of Selection in Jiaxian Red Cattle Using Whole-Genome Sequencing Data. BMC Genom..

[36] Liu X., Liu T., Wang Y., Dong H., Li F., Qi X., Luo Y., Jiang Y., Ahmed Z., Lei C. (2025). Genomic Ancestry and Adaptive Signatures in the Indigenous Hetian Cattle from Xinjiang Province of China Revealed by Whole-Genome Sequencing. BMC Genom..

[37] Hossein Banabazi M., Van Borm S., Klingström T., Niazi A., De Clercq K., Mostin L., Haegeman A., de Koning D.J. (2025). Host Transcriptome Profiling for Resistance against Lumpy Skin Disease (LSD). BMC Res. Notes.

[38] Chen Z., Yu M., Yan J., Guo L., Zhang B., Liu S., Lei J., Zhang W., Zhou B., Gao J. (2021). PNOC Expressed by B Cells in Cholangiocarcinoma Was Survival Related and LAIR2 Could Be a T Cell Exhaustion Biomarker in Tumor Microenvironment: Characterization of Immune Microenvironment Combining Single-Cell and Bulk Sequencing Technology. Front. Immunol..

[39] Wang L., Yan X., Wu H., Wang F., Zhong Z., Zheng G., Xiao Q., Wu K., Na W. (2024). Selection Signal Analysis Reveals Hainan Yellow Cattle Are Being Selectively Bred for Heat Tolerance. Animals.

[40] Singh A., Verma A., Dutta G., Gowane G.R., Ludri A., Alex R. (2024). Functional Transcriptome Analysis Revealed Major Changes in Pathways Affecting Systems Biology of Tharparkar Cattle under Seasonal Heat Stress. 3 Biotech.

[41] Yuan Y., Wei X., Xiong X., Wang X., Jiang W., Kuang Q., Zhu K., Chen C., Gan J., Li J. (2024). STAP2 Promotes the Progression of Renal Fibrosis via HSP27. J. Transl. Med..

[42] Marete A.G., Guldbrandtsen B., Lund M.S., Fritz S., Sahana G., Boichard D. (2018). A Meta-Analysis Including Pre-Selected Sequence Variants Associated With Seven Traits in Three French Dairy Cattle Populations. Front. Genet..

[43] Chen Z., Yao Y., Ma P., Wang Q., Pan Y. (2018). Haplotype-Based Genome-Wide Association Study Identifies Loci and Candidate Genes for Milk Yield in Holsteins. PLoS ONE.

[44] Chen Z., Chu S., Liang Y., Xu T., Sun Y., Li M., Zhang H., Wang X., Mao Y., Loor J.J. (2020). miR-497 Regulates Fatty Acid Synthesis via LATS2 in Bovine Mammary Epithelial Cells. Food Funct..

[45] Zhang D., Wang H., Chen Y., Cai Z., Yu B., Liu J., Feng X., Wang C., Gu Y., Zhang J. (2024). MicroRNA-2285f Regulates Milk Fat Metabolism by Targeting MAP2K2 in Bovine Mammary Epithelial Cells. Reprod. Domest. Anim..

[46] Vani S., Balasubramanyam D., Tirumurugaan K.G., Gopinathan A., Karthickeyan S.M.K. (2025). Genome-Wide Copy Number Variation Regions in Indigenous (*Bos indicus*) Cattle Breeds of Tamil Nadu, India. Anim. Biosci..

[47] Lee S.-H., Lee D., Lee M., Ryoo S.-H., Seo S., Choi I. (2022). Analysis of Single Nucleotide Polymorphisms Related to Heifer Fertility in Hanwoo (Korean Cattle). Anim. Biotechnol..

[48] Demir E., Moravčíková N., Kaya S., Kasarda R., Bilginer Ü., Doğru H., Balcıoğlu M.S., Karslı T. (2023). Genome-Wide Screening for Selection Signatures in Native and Cosmopolitan Cattle Breeds Reared in Türkiye. Anim. Genet..

[49] Frezarim G.B., Fonseca L.F.S., Salatta B.M., Silva D.B.S., Bresolin T., de Oliveira Seno L., Barufatti A., Ferro J.A., Albuquerque L.G. (2022). Genes and Proteins Associated with Ribeye Area and Meat Tenderness in a Commercial Nellore Cattle Population. Genome.

[50] Malheiros J.M., Enríquez-Valencia C.E., da Silva Duran B.O., de Paula T.G., Curi R.A., de Vasconcelos Silva J.A.I.I., Dal-Pai-Silva M., de Oliveira H.N., Chardulo L.A.L. (2018). Association of CAST2, HSP90AA1, DNAJA1 and HSPB1 Genes with Meat Tenderness in Nellore Cattle. Meat Sci..

[51] Silva E.F.P., Gaia R.C., Mulim H.A., Pinto L.F.B., Iung L.H.S., Brito L.F., Pedrosa V.B. (2024). Genome-Wide Association Study of Conformation Traits in Brazilian Holstein Cattle. Animals.

[52] Sheng H., Zhang J., Li F., Pan C., Yang M., Liu Y., Cai B., Zhang L., Ma Y. (2023). Genome-Wide Identification and Characterization of Bovine Fibroblast Growth Factor (FGF) Gene and Its Expression during Adipocyte Differentiation. Int. J. Mol. Sci..

[53] Lai X., Lan X., Chen H., Wang X., Wang K., Wang M., Yu H., Zhao M. (2009). A Novel SNP of the Hesx1 Gene in Bovine and Its Associations with Average Daily Gain. Mol. Biol. Rep..

[54] Rezende F.M., Dietsch G.O., Peñagaricano F. (2018). Genetic Dissection of Bull Fertility in US Jersey Dairy Cattle. Anim. Genet..

[55] Uzzaman M.R., Edea Z., Bhuiyan M.S.A., Walker J., Bhuiyan A.K.F.H., Kim K.-S. (2014). Genome-Wide Single Nucleotide Polymorphism Analyses Reveal Genetic Diversity and Structure of Wild and Domestic Cattle in Bangladesh. Asian-Australas. J. Anim. Sci..

[56] Barbato M., Reichel M.P., Passamonti M., Low W.Y., Colli L., Tearle R., Williams J.L., Ajmone Marsan P. (2020). A Genetically Unique Chinese Cattle Population Shows Evidence of Common Ancestry with Wild Species When Analysed with a Reduced Ascertainment Bias SNP Panel. PLoS ONE.

[57] Strucken E.M., Gebrehiwot N.Z., Swaminathan M., Joshi S., Al Kalaldeh M., Gibson J.P. (2021). Genetic Diversity and Effective Population Sizes of Thirteen Indian Cattle Breeds. Genet. Sel. Evol..

[58] Liu Y., Xu L., Yang L., Zhao G., Li J., Liu D., Li Y. (2020). Discovery of Genomic Characteristics and Selection Signatures in Southern Chinese Local Cattle. Front. Genet..

[59] Wang P., Ou G., Li G., Li H., Zhao T. (2024). Analysis of Genetic Diversity and Structure of Endangered Dengchuan Cattle Population Using a Single-Nucleotide Polymorphism Chip. Anim. Biotechnol..

[60] Gao Y., Gautier M., Ding X., Zhang H., Wang Y., Wang X., Faruque M.O., Li J., Ye S., Gou X. (2017). Species Composition and Environmental Adaptation of Indigenous Chinese Cattle. Sci. Rep..

[61] Du J., Yang Y., Wei D., Wu J., Tian C., Hu Q., Bian H., Cheng C., Zhai X. (2025). The Role of DUOXA2 in the Clinical Diagnosis of Paediatric Congenital Hypothyroidism. Ann. Med..

[62] Liu S., Han W., Zang Y., Zang H., Wang F., Jiang P., Wei H., Liu X., Wang Y., Ma X. (2019). Identification of Two Missense Mutations in DUOX1 (p.R1307Q) and DUOXA1 (p.R56W) That Can Cause Congenital Hypothyroidism Through Impairing H_2_O_2_ Generation. Front. Endocrinol..

[63] Nikanorova A., Barashkov N., Pshennikova V., Teryutin F., Nakhodkin S., Solovyev A., Romanov G., Burtseva T., Fedorova S. (2023). A Systematic Review and Meta-Analysis of Free Triiodothyronine (FT3) Levels in Humans Depending on Seasonal Air Temperature Changes: Is the Variation in FT3 Levels Related to Nonshivering Thermogenesis?. Int. J. Mol. Sci..

[64] Mota-Rojas D., Ghezzi M.D., Hernández-Ávalos I., Domínguez-Oliva A., Casas-Alvarado A., Lendez P.A., Ceriani M.C., Wang D. (2024). Hypothalamic Neuromodulation of Hypothermia in Domestic Animals. Animals.

[65] Zhou H., Cheng Z., Bass N., Krystal J.H., Farrer L.A., Kranzler H.R., Gelernter J. (2018). Genome-Wide Association Study Identifies Glutamate Ionotropic Receptor GRIA4 as a Risk Gene for Comorbid Nicotine Dependence and Major Depression. Transl. Psychiatry.

[66] (2024). Identification of a Cold Sensor in Peripheral Somatosensory Neurons. Nat. Neurosci..

[67] Gong J., Liu J., Ronan E.A., He F., Cai W., Fatima M., Zhang W., Lee H., Li Z., Kim G.-H. (2019). A Cold-Sensing Receptor Encoded by a Glutamate Receptor Gene. Cell.

[68] Zhou Y., Ren W., Shao W., Gao Y., Yao K., Yang M., Zhang X., Wang Y., Li F., Yang L. (2025). Exploration of Non-Coding RNAs Related to Intramuscular Fat Deposition Xinjiang Brown Cattle and Angus × Wagyu Cattle. BMC Genom..

[69] Lee J., Jeong T., Park W., Jang S., Lee P.-Y., Lim D. (2025). Identification of Expression Quantitative Trait Loci (eQTL) for Adipose-Specific Regulatory Mechanisms in Hanwoo (Korean Cattle). Animals.

[70] Zhou J., Li L., Wu B., Feng Z., Lu Y., Wang Z. (2024). MST1/2: Important Regulators of Hippo Pathway in Immune System Associated Diseases. Cancer Lett..

[71] Tang D., Xu H., Du X. (2023). The Role of Non-Canonical Hippo Pathway in Regulating Immune Homeostasis. Eur. J. Med. Res..

[72] Zhong Z., Jiao Z., Yu F.-X. (2024). The Hippo Signaling Pathway in Development and Regeneration. Cell Rep..

[73] St Louis B.M., Quagliato S.M., Su Y.-T., Dyson G., Lee P.-C. (2024). The Hippo Kinases Control Inflammatory Hippo Signaling and Restrict Bacterial Infection in Phagocytes. mBio.

[74] Aboul-Naga A.M., Alsamman A.M., El Allali A., Elshafie M.H., Abdelal E.S., Abdelkhalek T.M., Abdelsabour T.H., Mohamed L.G., Hamwieh A. (2022). Genome-Wide Analysis Identified Candidate Variants and Genes Associated with Heat Stress Adaptation in Egyptian Sheep Breeds. Front. Genet..

[75] Qi F., Isaji T., Duan C., Yang J., Wang Y., Fukuda T., Gu J. (2020). ST3GAL3, ST3GAL4, and ST3GAL6 Differ in Their Regulation of Biological Functions via the Specificities for the A2,3-Sialylation of Target Proteins. FASEB J..

[76] Demirel A.F., Çak B. (2025). Associations Between Polymorphisms of the CSN1S1, CSN1S2, CSN2 and CSN3 Genes and Milk Composition Traits in Holstein Cattle. Vet. Med. Sci..

[77] Younis A., Hussain I., Ahmad S.N., Shah A., Inayat I., Kanwal M.A., Suleman S., Kamran M.A., Matloob S., Ahmad K.R. (2024). Validation of *Bos Taurus* SNPs for Milk Productivity of Sahiwal Breed (*Bos indicus*), Pakistan. Animals.

[78] Igoshin A.V., Yudin N.S., Belonogova N.M., Larkin D.M. (2019). Genome-Wide Association Study for Body Weight in Cattle Populations from Siberia. Anim. Genet..

[79] Wu X., Fang M., Liu L., Wang S., Liu J., Ding X., Zhang S., Zhang Q., Zhang Y., Qiao L. (2013). Genome Wide Association Studies for Body Conformation Traits in the Chinese Holstein Cattle Population. BMC Genom..

[80] Alipoor S.D., Mirsaeidi M. (2021). Inborn Errors in the LRR Domain of Nod2 and Their Potential Consequences on the Function of the Receptor. Cells.

[81] Lu X., Arbab A.A.I., Abdalla I.M., Liu D., Zhang Z., Xu T., Su G., Yang Z. (2021). Genetic Parameter Estimation and Genome-Wide Association Study-Based Loci Identification of Milk-Related Traits in Chinese Holstein. Front. Genet..

[82] Jin L., Qu K., Hanif Q., Zhang J., Liu J., Chen N., Suolang Q., Lei C., Huang B. (2022). Whole-Genome Sequencing of Endangered Dengchuan Cattle Reveals Its Genomic Diversity and Selection Signatures. Front. Genet..

[83] Xia W., Osorio J.S., Yang Y., Liu D., Jiang M.F. (2018). Short Communication: Characterization of Gene Expression Profiles Related to Yak Milk Protein Synthesis during the Lactation Cycle. J. Dairy Sci..

[84] Fu L., Zhang L., Liu L., Yang H., Zhou P., Song F., Dong G., Chen J., Wang G., Dong X. (2021). Effect of Heat Stress on Bovine Mammary Cellular Metabolites and Gene Transcription Related to Amino Acid Metabolism, Amino Acid Transportation and Mammalian Target of Rapamycin (mTOR) Signaling. Animals.

[85] Li Y., Li S., Zhao X., Shi C., Chai Y., Huang A., Shi Y. (2024). Novel Insights into Whey Protein among Yak, Yellow Cattle, and Cattle-Yak Milk. Food Chem. X.

[86] Fulford A.D., McNeill H. (2020). Fat/Dachsous Family Cadherins in Cell and Tissue Organisation. Curr. Opin. Cell Biol..

[87] Wang X., Pu Y., Miao J., Xie L., Guan L., Cui Y., Wang J., Qin L., Han Y., Wöhr M. (2025). Atypical Cadherin FAT2 Is Required for Synaptic Integrity and Motor Behaviors. J. Neurosci..

[88] Knapp B., Wolfrum U. (2016). Adhesion GPCR-Related Protein Networks. Handb. Exp. Pharmacol..

[89] Li R., Chen S., Li C., Xiao H., Costa V., Bhuiyan M.S.A., Baig M., Beja-Pereira A. (2022). Whole-Genome Analysis Deciphers Population Structure and Genetic Introgression Among Bovine Species. Front. Genet..

[90] Dai X., Bian P., Hu D., Luo F., Huang Y., Jiao S., Wang X., Gong M., Li R., Cai Y. (2023). A Chinese Indicine Pangenome Reveals a Wealth of Novel Structural Variants Introgressed from Other Bos Species. Genome Res..

[91] Koyanagi M., Nakabayashi K., Fujimoto T., Gu N., Baba I., Takashima Y., Doi K., Harada H., Kato N., Sasazuki T. (2008). ZFAT Expression in B and T Lymphocytes and Identification of ZFAT-Regulated Genes. Genomics.

[92] Costilla R., Kemper K.E., Byrne E.M., Porto-Neto L.R., Carvalheiro R., Purfield D.C., Doyle J.L., Berry D.P., Moore S.S., Wray N.R. (2020). Genetic Control of Temperament Traits across Species: Association of Autism Spectrum Disorder Risk Genes with Cattle Temperament. Genet. Sel. Evol..

[93] Wang Y.-N., Tang Y., He Z., Ma H., Wang L., Liu Y., Yang Q., Pan D., Zhu C., Qian S. (2021). Slit3 Secreted from M2-like Macrophages Increases Sympathetic Activity and Thermogenesis in Adipose Tissue. Nat. Metab..

[94] Serdan T.D.A., Frank B., Cervantes H., Gargey A., Tian Q., Hope D., Choi C.H.J., Hoffmann A., Cohen P., Blüher M. (2026). Slit3 Fragments Orchestrate Neurovascular Expansion and Thermogenesis in Brown Adipose Tissue. Nat. Commun..

[95] Chi Z., Jia Q., Yang H., Ren H., Jin C., He J., Wuri N., Sui Z., Zhang J., Mengke B. (2024). snRNA-Seq of Adipose Tissues Reveals the Potential Cellular and Molecular Mechanisms of Cold and Disease Resistance in Mongolian Cattle. BMC Genom..

[96] Wang J., Shen N., Zhao K., Liao J., Jiang G., Xiao J., Jia X., Sun W., Lai S. (2025). Revealing Study and Breeding Implications for Production Traits and Tail Characteristics in Simmental Cattle by GWAS. Front. Genet..

[97] Meng G., Bao Q., Ma X., Chu M., Huang C., Guo X., Liang C., Yan P. (2022). Analysis of Copy Number Variation in the Whole Genome of Normal-Haired and Long-Haired Tianzhu White Yaks. Genes.

[98] Yang C., Yang Y., Zhao B., Gao E., Chen H., Li Y., Ma J., Wang J., Hu S., Song X. (2024). Comparative Analysis of Differentially Expressed Genes and Transcripts in the Ovary of Yak in Estrus and Anestrus. Anim. Biotechnol..

[99] Chen X., Duan X., Chong Q., Li C., Xiao H., Chen S. (2023). Genome-Wide DNA Methylation Differences between *Bos indicus* and *Bos Taurus*. Animals.

