# Supplementary material for: Genetic Diversification and Population Admixture Signatures in Yunnan Native Cattle
Source: Animals (Basel). 2026 Apr 3;16(7):1105. doi: 10.3390/ani16071105 (PMC13072361; doi:10.3390/ani16071105)
Supplement: Supplementary file 1 [file animals-16-01105-s001.zip › Figures S1-S10.pdf]

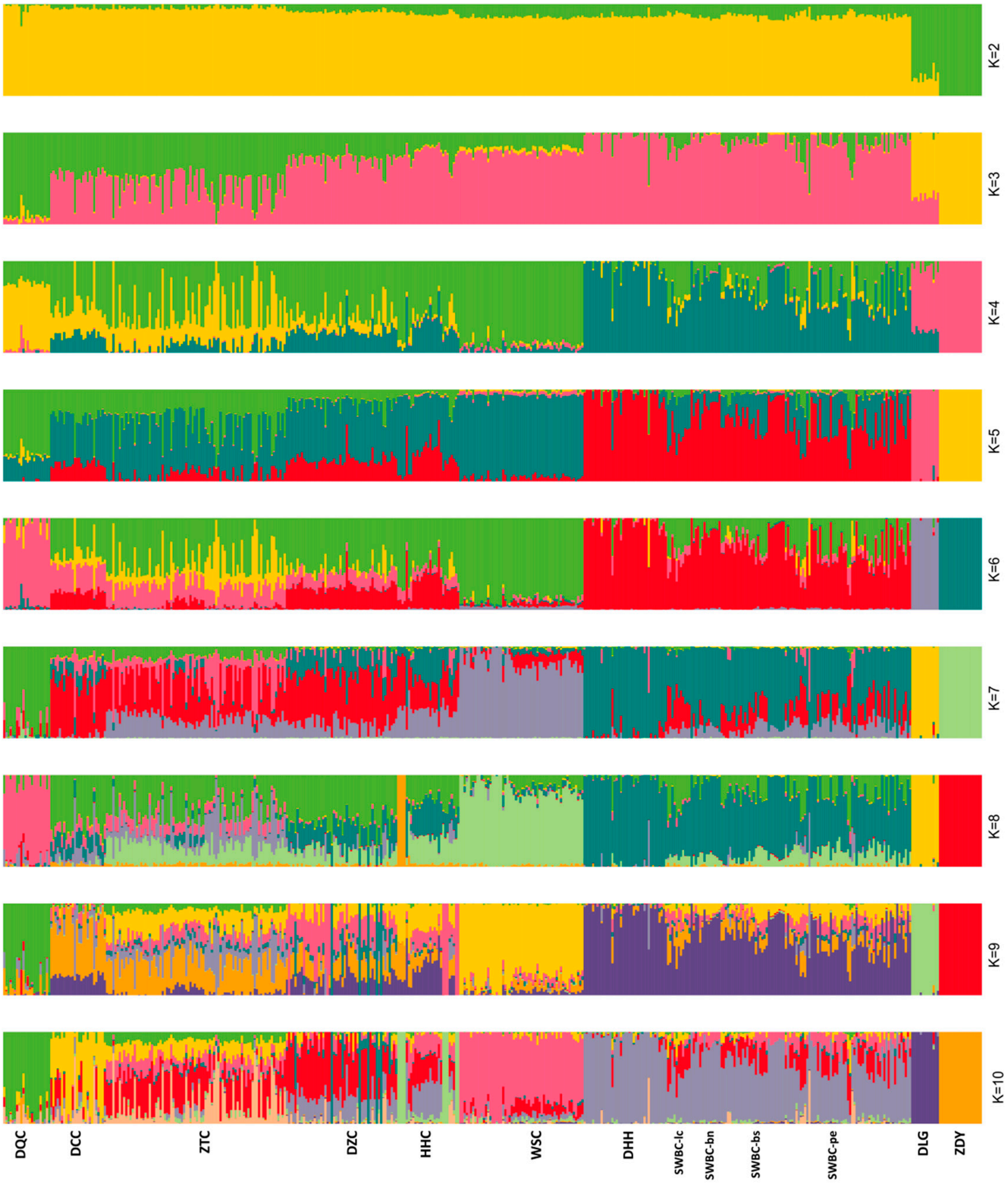

Figure S1. Admixture patterns inferred for K = 2 to 10 within Yunnan bovine populations, providing a detailed view of sub-population divergence across a wider range of ancestral components.

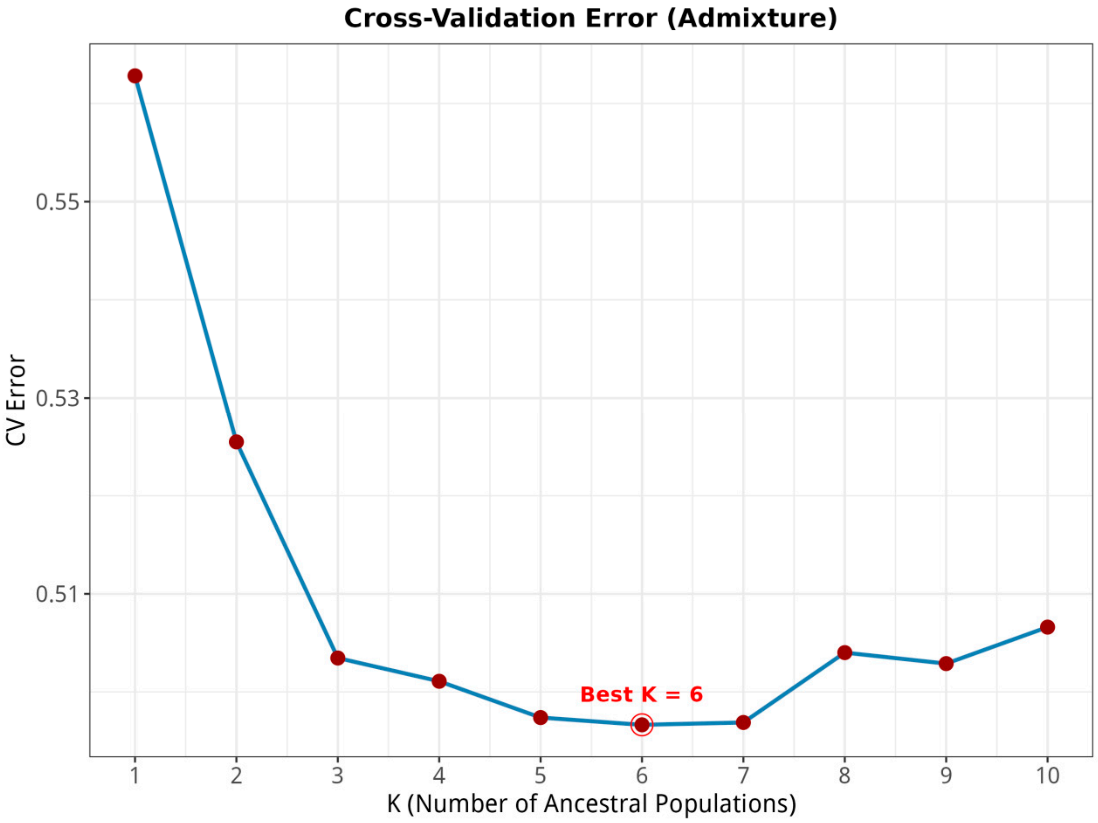

Figure S2. Cross-validation (CV) error plot for different numbers of ancestral populations (K) in ADMIXTURE analysis. (K=6, CV=0.496)

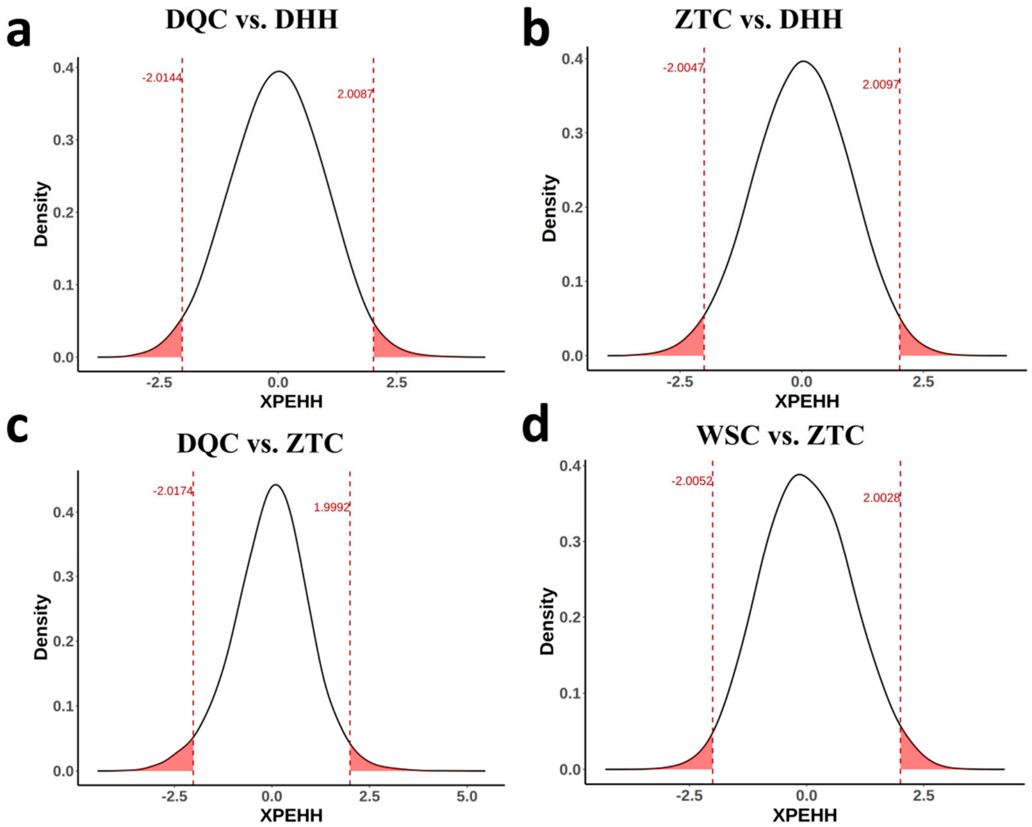

Figure S3. Genome-wide distribution and empirical thresholding of normalized XPEHH scores. Density plots of normalized XPEHH scores for (a) DQC vs. DHH; (b) ZTC vs. DHH; (c) DQC vs. ZTC; and (d) WSC vs. ZTC. Red dashed lines denote the empirical significance thresholds ($p < 0.01$, two-tailed) used to identify outlier SNPs.

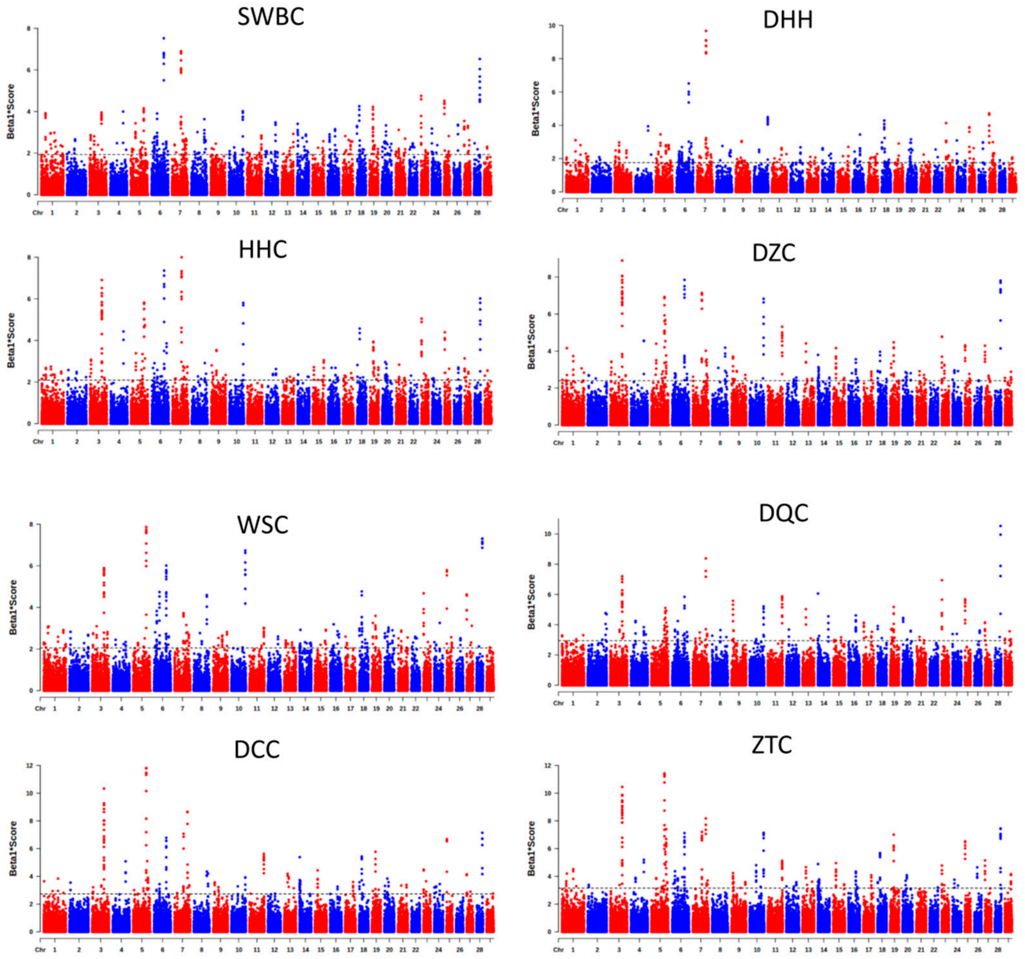

Figure S4. Manhattan plot of BetaScan2 scores for balancing selection across eight YNC populations. Cattle in the southwest border of Yunnan (SWBC); Dehong humped cattle (DHH); Honghe cattle (HHC); Dianzhong cattle (DZC); Wenshan cattle (WSC); Diqing cattle (DQC); Dengchuan cattle (DCC); Zhaotong cattle (ZTC).

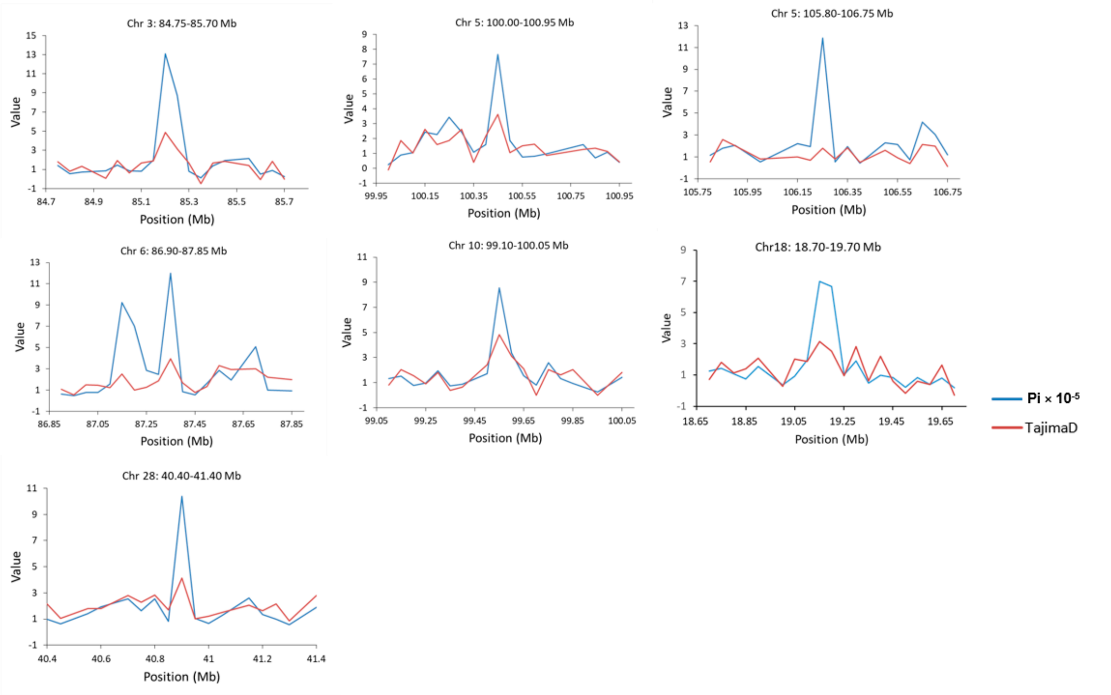

Figure S5. Local patterns of nucleotide diversity (Pi) and Tajima's D across candidate balancing selection regions.

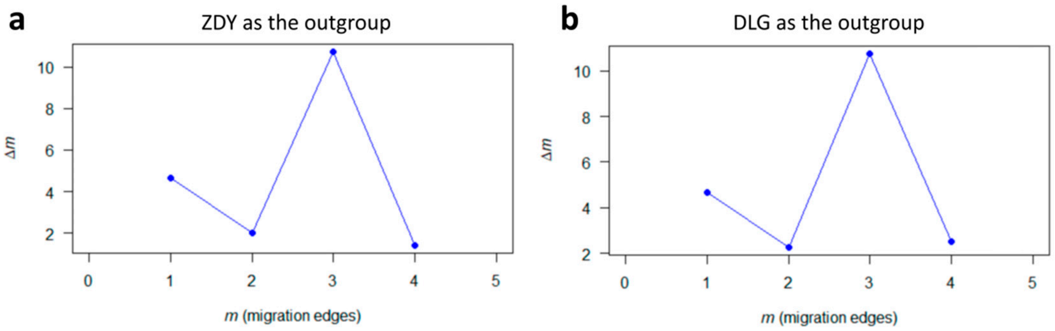

Figure S6. Δ m values for different numbers of migration edges (m) in TreeMix analysis, using (a) ZDY and (b) DLG as outgroups.

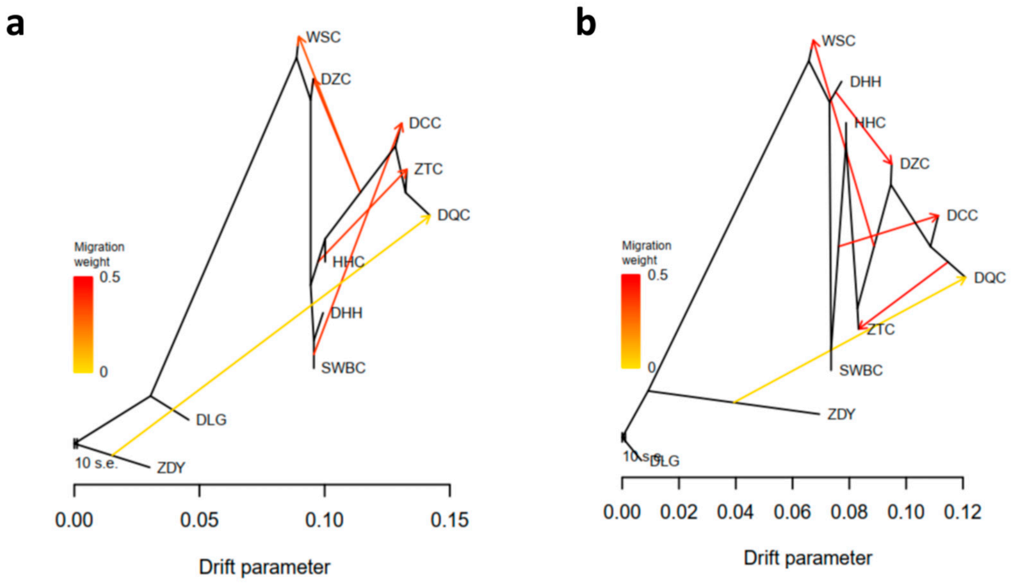

Figure S7. Maximum likelihood trees inferred by TreeMix with 5 migration edges, using ZDY (left) and DLG (right) as the outgroup.

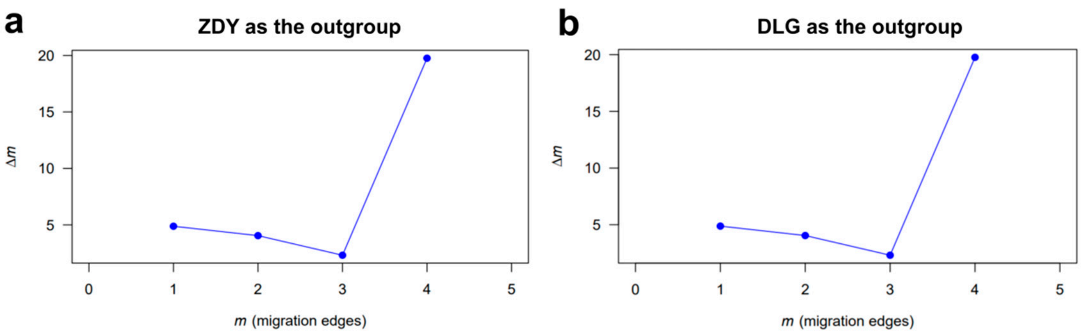

Figure S8. Δ m values for different numbers of migration edges (m) in TreeMix analysis, using (a) ZDY and (b) DLG as outgroups. TreeMix analysis performed using a randomly down-sampled SWBC population (n = 25) to account for sample size asymmetry.

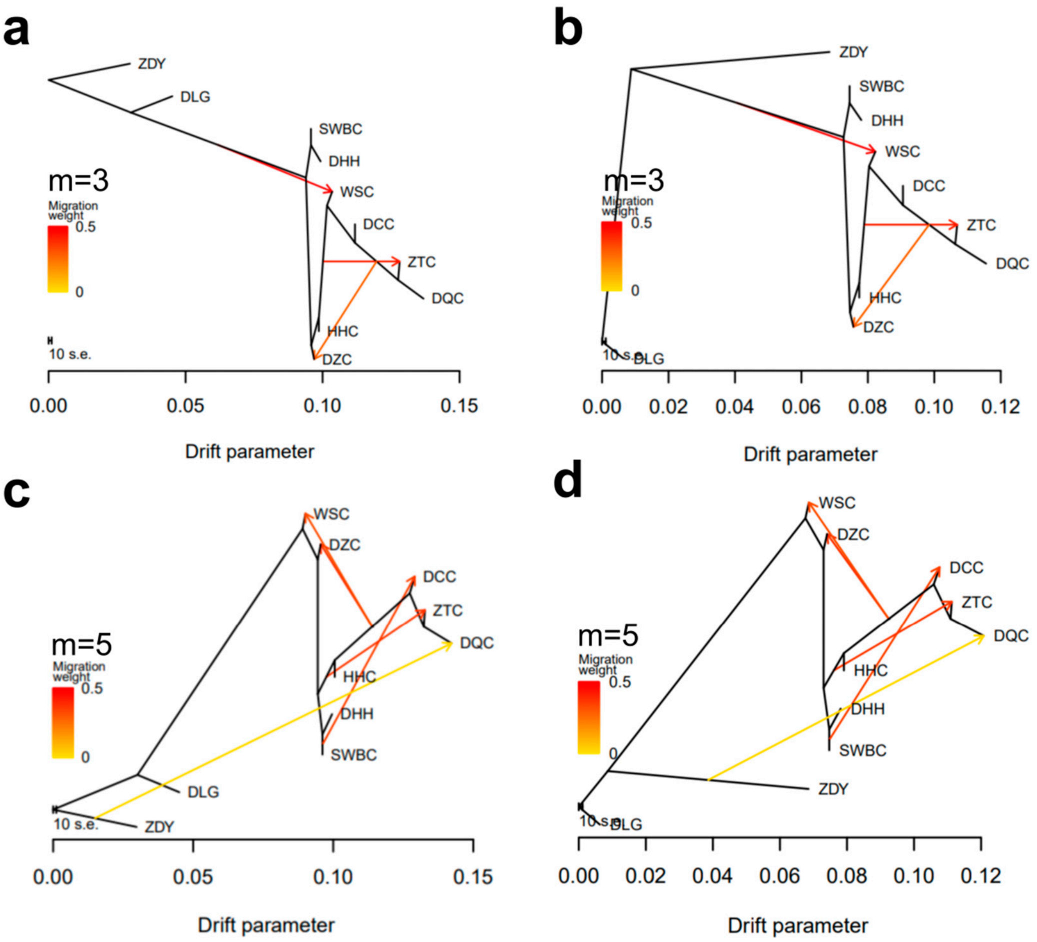

Figure S9. Maximum likelihood trees inferred by TreeMix with (a,b) 3 and (c,d) 5 migration edges, using ZDY (a,c) and DLG (b,d) as the outgroup. TreeMix analysis performed using a randomly down-sampled SWBC population (n = 25) to account for sample size asymmetry.

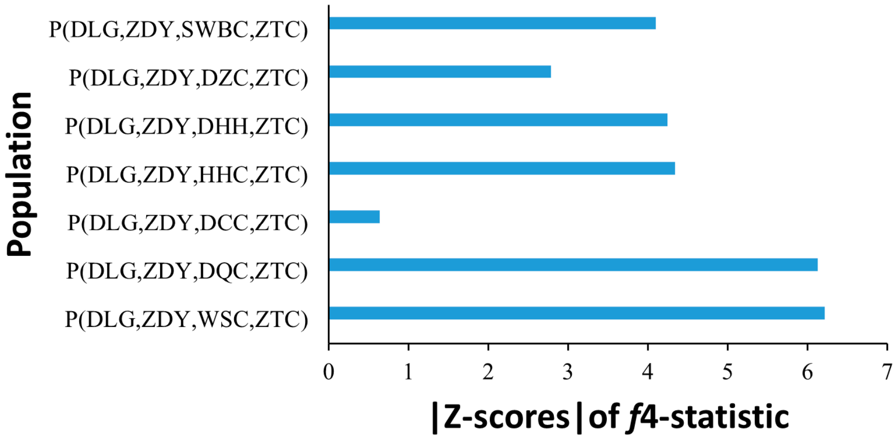

Figure S10. |Z-scores| of the *f4*-statistics under the (DLG, ZDY; X, ZTC) topology. Analysis of *f4*-statistics performed using a randomly down-sampled SWBC population (n = 25) to account for sample size asymmetry.
